# Clinical Course of Pediatric Acute Respiratory Distress Syndrome at Moderate Altitude

**DOI:** 10.7759/cureus.10651

**Published:** 2020-09-25

**Authors:** María A Chacón, Alejandra Calderon, Jaime Fernández-Sarmiento, Blanca Rios

**Affiliations:** 1 Pediatric Critical Care, Universidad de La Sabana, Bogotá, COL; 2 Pediatric Oncology, Instituto Nacional de Cancerologia, Bogotá, COL; 3 Pediatric Critical Care, Fundación Cardioinfantil Instituto de Cardiología, Bogotá, COL

**Keywords:** acute respiratory distress syndrome, children, protective ventilation, moderate altitude

## Abstract

Background

This is a retrospective case series, and the main objective is to describe the epidemiology, clinical features, and outcomes of pediatric acute respiratory distress syndrome in patients at moderate altitude.

Methods

Children from the Pediatric Intensive Care Unit (PICU) at the Fundación Cardioinfantil, hospitalized with acute respiratory distress syndrome, were prospectively enrolled from March 2009 to March 2014. We evaluated the demographic data, mechanical ventilation, gas exchange, hemodynamics, and multiorgan dysfunction.

Results

During the study period, 88 patients met the inclusion criteria. Bronchiolitis and pneumonia were the most common causes of acute respiratory distress syndrome. The overall mortality rate was 19.5%. At the beginning of the study, the average relation between blood pressure and the fraction of inspired oxygen (Pa/Fi) was 130.3 ± 52.2; tidal volume was 7.94 ± 1.7 ml/kg, the plateau pressure 25.3 ± 5.09 cmH_2_O, and positive end-expiratory pressure was 7.2 ± 3.2 cmH_2_O. After 24 hours, the mortality rate in the group with severe acute respiratory distress syndrome (Pa/Fi <100) was 46.7%, in the moderate acute respiratory distress syndrome group (Pa/Fi 100-200) it was 11.9%, and finally in the mild acute respiratory distress syndrome group (Pa/Fi 200-300) the mortality was 25%. This study found a relation between serum lactate value and positive end-expiratory pressure and mortality (p = 0.02 and 0.0013).

Conclusions

This study shows that pediatric acute respiratory distress syndrome patients at moderate altitudes have similar clinical behavior, including mortality rate, to those at low altitudes. However, Pa/Fi is not a good predictor of mortality for patients with mild and moderate acute respiratory distress syndrome.

## Introduction

Acute respiratory distress syndrome (ARDS) is characterized by injury of the alveolar-capillary membrane leading to pulmonary edema, decreased pulmonary compliance, and increased shunt, causing an impaired oxygenation/ventilation and respiratory failure [1].

ARDS was first described in 1967 by Dr. Ashbaug in a case series of 12 patients with tachypnea, cyanosis, severe dyspnea refractory to oxygen, loss of pulmonary compliance, and patchy alveolar infiltrates on chest x-ray [2]. Later in 1994, the American European Consensus established the main components of the definition including chest x-ray and the relation between blood pressure and fraction of inspired oxygen (Pa/Fi), and according to those parameters categorized acute lung injury and ARDS [3].

Using a consensus process, a panel of experts convened in 2011 (an initiative of the European Society of Intensive Care Medicine endorsed by the American Thoracic Society and the Society of Critical Care Medicine) developed the Berlin Definition. With new components of the definition of ARDS including the positive end-expiratory pressure (PEEP), timing, and chest radiograph findings, they proposed three mutually exclusive categories of ARDS based on the degree of hypoxemia: mild (200 mmHg < Pa/Fi ≤ 300 mmHg), moderate (100 mmHg < Pa/Fi ≤ 200 mmHg), and severe (Pa/Fi ≤ 100 mmHg) [4].

The ARDS criteria have been described in the adult population with extrapolation of data to pediatric patients. Currently, there are few studies about epidemiology and mortality in children [1,4,5].

Lopez et al. reported an overall mortality rate of 28% in pediatric patients; however, the mortality rate may change according to the Pa/Fi, (38% in severe ARDS and 11% in moderate ARDS), and he described a linear relationship between the number of impaired organs and mortality - when more than three organs are impaired the mortality increases to 70% [5].

Recent research has established the ARDS natural history in patients living below 1,000 metres above mean sea level (MAMSL) [5]. However, there are no clinical studies about ARDS natural history or mortality in cities above this level [6,7]. As the altitude increases, the availability of oxygen decreases, which is why humans have developed adaptive mechanisms to avoid hypoxemia [6,7]. Mild or moderate increases of altitude above 3000 meters are associated with important changes in oxygen saturation and pulmonary artery pressure. Altitude induces adaptive changes to the decrease in alveolar oxygen pressure [8]. 

The cardiovascular response is one of them; the hypoxia produces an initial increase in cardiac output by 22% and the heart rate by 18%, maintaining a constant systolic volume. Then the cardiac output returns to the standard value with a decrease in systolic volume and persistence of high heart rate. Additionally, there is a decrease in maximum oxygen uptake to maintain homeostasis. Another component of the cardiovascular response is activation of the sympathetic nervous system in response to hypoxia; studies show an increase in catecholamine levels in the blood and urine of these patients, causing the high heart rate and hemodynamic changes described [9].

After two weeks of exposure to hypoxemia, hemoglobin synthesis is increased and, therefore, the blood viscosity, which results in decreased cardiac output, in theory; however, studies using isovolumetric hemodilution do not increase peripheral oxygen uptake and only increase cardiac output slightly [9].

We believe it is essential to analyze the disease's clinical behavior and establish the outcome in children with ARDS who have developed physiological mechanisms of adaptation to living at moderate and high altitudes.

This research was conducted to determine the natural history and etiology of ARDS in the pediatric population in Bogotá, located 2,630 meters above mean sea level (moderate altitude) [10].

## Materials and methods

Study design and patients

This observational retrospective case series was approved by the ethics committee of the Fundación Cardioinfantil Instituto de Cardiologia. It was not necessary to sign informed consent by parents because no intervention was performed on patients, and data was collected from the medical history.

During a period of five years (March 2009 to March 2014), patients aged from one month to 18 years admitted to the pediatric intensive care unit (PICU) of the Fundación Cardioinfantil Instituto de Cardiologia, who met the inclusion criteria based on the consensus of Berlin [2] were included in this study: respiratory symptoms within one week of a known clinical insult or new or worsening, chest x-ray with bilateral opacities not fully explained by effusions, lobar/lung collapse or nodules, edema not fully explained by cardiac failure or fluid overload, Pa/Fi ≤ 300 mmHg.

Exclusion criteria were: patients under one month and over 18 years, patients with congenital heart disease, and patients with cardiogenic pulmonary edema.

Data collection and quality control

**Figure 1:**
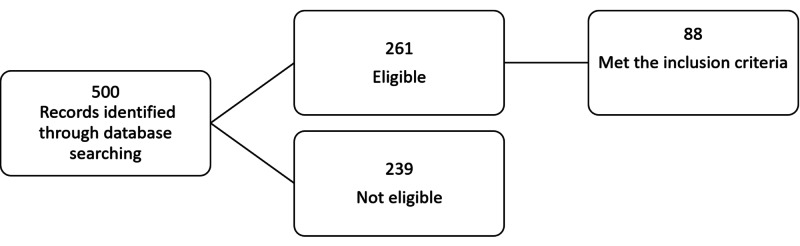
Patients flow diagram.

In the beginning, data was collected from all patients admitted to the PICU at the Fundación Cardioinfantil from March 2009 to March 2014, whose admission diagnoses were pneumonia, bronchiolitis, respiratory failure, and septic shock. After this initial screening, these medical records were reviewed, and the inclusion and exclusion criteria were applied. Data were included in the study through a data collection instrument (Figure 1).

The onset of ARDS was defined as when the patient met the inclusion criteria set forth above. Demographic data, hemodynamic, pediatric index of mortality (PIM 2), arterial blood gases, ventilatory parameters and modes, chest x-ray, serum lactate, hemoglobin, and comorbidities were included (pneumothorax, shock septic, and multiorgan failure).

Plasma lactate values, hemodynamic data, ventilator settings, arterial blood gases, and hemoglobin were analyzed at the time of the ARDS diagnosis, at 24 hours, day three, day seven, and the last day of mechanical ventilation.

The relation between Pa/Fi and PEEP levels of more than 5 cmH_2_O for classifying ARDS in mild, moderate, and severe was used: mild (200 mmHg < Pa/Fi ≤ 300 mmHg), moderate (100 mmHg < Pa/Fi ≤ 200 mmHg), and severe (Pa/Fi ≤ 100 mmHg). Pa/Fi ratio, septic shock, multiple organ dysfunction, and pneumothorax were also evaluated.

The ventilation parameters tidal volume in ml/kg, PEEP, and plateau pressure were analyzed; in high-frequency ventilation, the parameters mean airway pressure, amplitude, respiratory rate, and the fraction of inspired oxygen (FIO_2_) were analyzed. Ventilation modes were also identified. Total days of mechanical ventilation, length of hospital stay, and mortality rate were analyzed.

Statistic analysis

Absolute and relative frequencies for categorical variables, and the median and interquartile range for quantitative variables before assessing the normality assumption based on the Shapiro Wilks was obtained. The correlation between mortality and severity, the number of involved organs, tidal volume, PEEP, and serum lactate was made by Spearman's Rank Correlation Coefficient.

The comparison between the medians of the variables tidal volume, PEEP, and serum lactate at 24 hours and last day of mechanical ventilation between the group of patients who died and those who did not, was done using the Mann-Whitney U test. All statistical analyses were performed using a standard software package (Stata version 12; StataCorp., College Station, TX, USA).

## Results

During the study, 500 medical records were reviewed, and 261 of them were eligible because of the diagnosis at the admission of shock, ventilatory failure, pneumonia, or bronchiolitis. Only 88 patients met the inclusion criteria (Figure 1). The median age was one year, and 59.8% were men. The leading cause of ARDS was pneumonia in 78.1%, followed by bronchiolitis in 9.1% and sepsis in third place. Septic shock was present in 97.7% of cases. The overall mortality rate was 19.5%, and the Pediatric Mortality Index 2 at admission was 2 ± 1.3; the last one was used as a severity score (Table 1).

**Table 1:** Demographic characteristics of patients and clinical details at diagnosis of ARDS. Max: Maximum; Min: Minimum; n: Number of assessed children; SD: Standard deviation. ARDS: Acute Respiratory Distress Syndrome, PEEP: Positive End-Expiratory Pressure, Pa/Fi: Relation between blood pressure and the fraction of inspired oxygen, PIM2: Pediatric Index of Mortality score 2

Parameter	n	%	Median	Min	Max	Mean	SD
Age (years)			1	0.4	4		
Sex							
Male	156	59.8					
Female	105	40.2					
Length of stay in PICU (days)			15	10	23		
Mechanical ventilation days			8	6	14		
Pa/Fi at admission						130.3	52.2
Tidal volume (ml/kg) Onset of ARDS						7.94	1.7
Plateau pressure (cmH_2_O) Onset ARDS						25.3	5.09
PEEP (cmH_2_O) Onset ARDS						7.2	3.2
Neumothorax	5	5.7					
Septic shock	85	97.7					
Multiorgan dysfunction	71	81					
Number of organs involved							
Kidney – Heart- Lung- Hematologic	22	25					
Kidney – Heart- Lung	32	36.7					
Heart- Lung	17	19.5					
Mortality	17	19.5					
Pediatric Index of Mortality (PIM2) score 2						2	1.3
Causes of ARDS							
Pneumonia	68	78.1					
Bronchiolitis	8	9.1					
Sepsis	7	8					
Others	17	4.8					

The most common ventilation modes used at the beginning of ARDS were control pressure in 58.6% of the cases, followed by pressure regulated volume control in 21.8% of the cases. Weaning from mechanical ventilation was performed using Duopap in 24.1% of the cases, followed by Synchronized Intermittent Mandatory Ventilation (SIMV) mode with Pressure Support Ventilation (PSV) in 13.7% of the cases.

High-frequency ventilation was used as the initial ventilatory mode in three patients (3.4%), and it was used for 16 patients (18.3%) over the development of ARDS.

At the diagnosis of ARDS, the tidal volume was 7.9 ml/kg ± 1.7 ml/kg, the median was maintained between 7 ml/kg and 8 ml/kg throughout the study. The plateau pressure baseline was 25.3 ± 5.09 cmH_2_O and varied from 15 cmH_2_O to 26 cmH_2_O. PEEP was 7.2 cmH_2_O ± 3.2 cmH_2_O, and the median was maintained between 6 cmH_2_O and 6.5 cmH_2_O. That was the lung-protective ventilation strategy used in the study. A detailed description of the ventilatory gas exchange variables throughout the study is reported in Table 2.

**Table 2:** Ventilatory gas exchange variables throughout the study. Values are presented in mean ± SD PaCO2: Partial Pressure of Carbon dioxide, PEEP: Positive End-Expiratory Pressure, Pa/Fi: Relation between blood pressure and the fraction of inspired oxygen

Variable	Diagnosis	24 hours	Day 3	Day 7	Last day of mechanical ventilation
Tidal volume (ml/kg)	7.90 ± 1.75	10.4±1.43	10.4± 10.4	8.11±1.43	8.26 ± 1.6
Plateau pressure (cmH_2_O)	25.3 ±5.09	25.3±5.79	23.7±5.85	22.1±6.45	16.77±7.32
PEEP (cm H_2_O)	7.20±3.24	6.78±2.22	6.55±2.37	7.22±2.92	5.6±3.67
Pa/Fi (mmHg)	130.33±52.3	150.4±53.5	152.3±70.06	144.6±47.5	206.6±89.6
pH	7.22 ±0.75	7.37±0.31	7.40±0.13	7.43±0.07	7.40±0.13
PaCO_2_ (mmHg)	48.67±18.7	46.9±17.96	44.2±14.74	44.16±12.61	41.5±16.63
Serum lactate (mmol/L)	2.10±3.41	1.29±0.93	1.36±1.26	1.17±1.26	1.59±1.67
Hemoglobin (g/dl)	10.40±1.89	10.07±1.50	10.46±1.39	10.26±1.60	10.58±1.72

The mean length of mechanical ventilation was 11.4 ± 8.2 days, and 39 patients (44.8%) were extubated during the first week of ARDS. The median length of stay in PICU was 15 (10-23) days.

**Figure 2:**
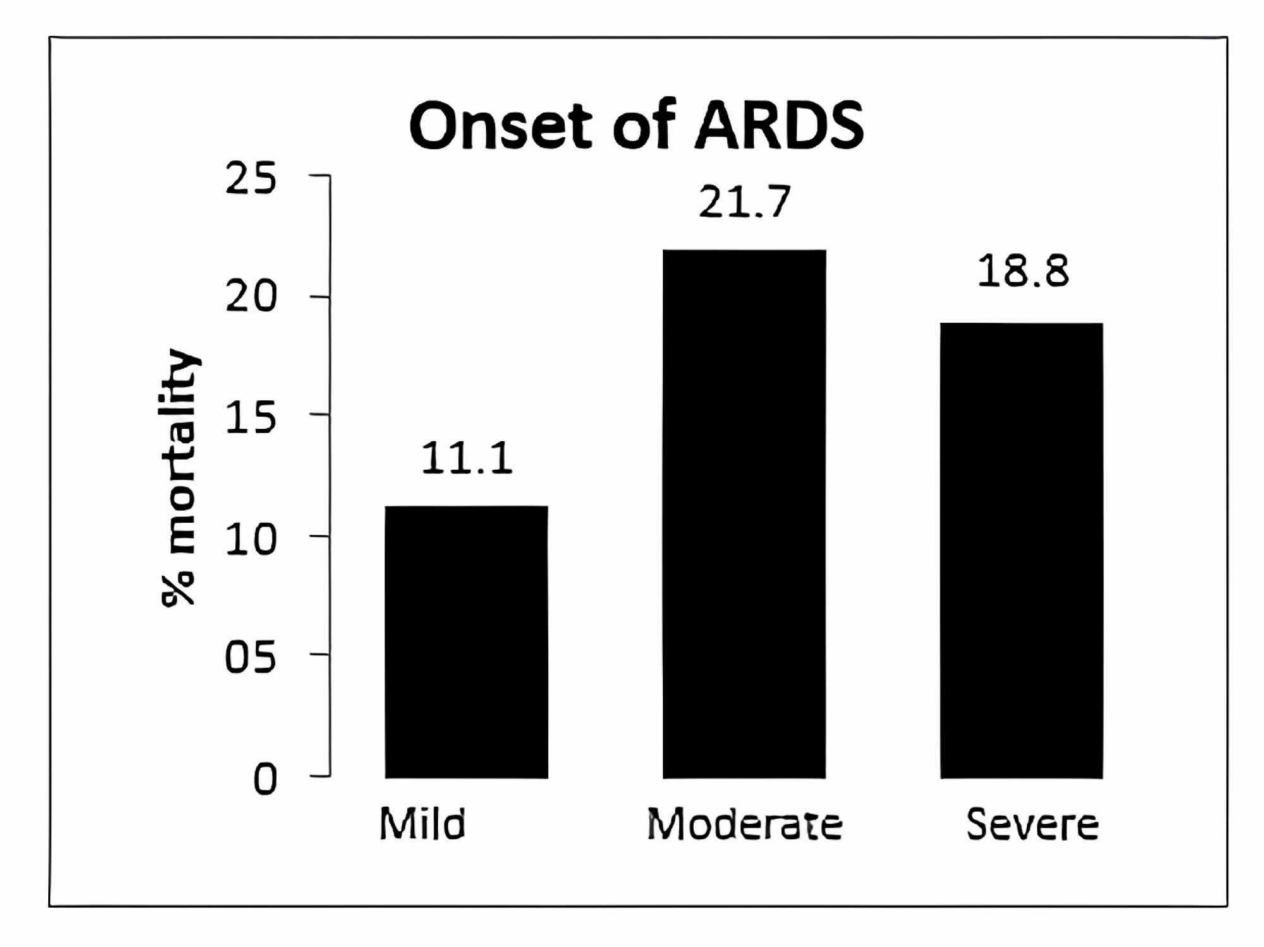
Mortality rate according to ARDS severity at diagnosis (based on the Pa/Fi ratio). ARDS: Acute Respiratory Distress Syndrome, Pa/Fi: Relation between blood pressure and the fraction of inspired oxygen

The mortality rate with ARDS varies based on severity. We found that patients at diagnosis with mild, moderate, and severe ARDS had mortality rates of 11%, 21.7%, and 18.8%, respectively (Figure 2).

**Figure 3:**
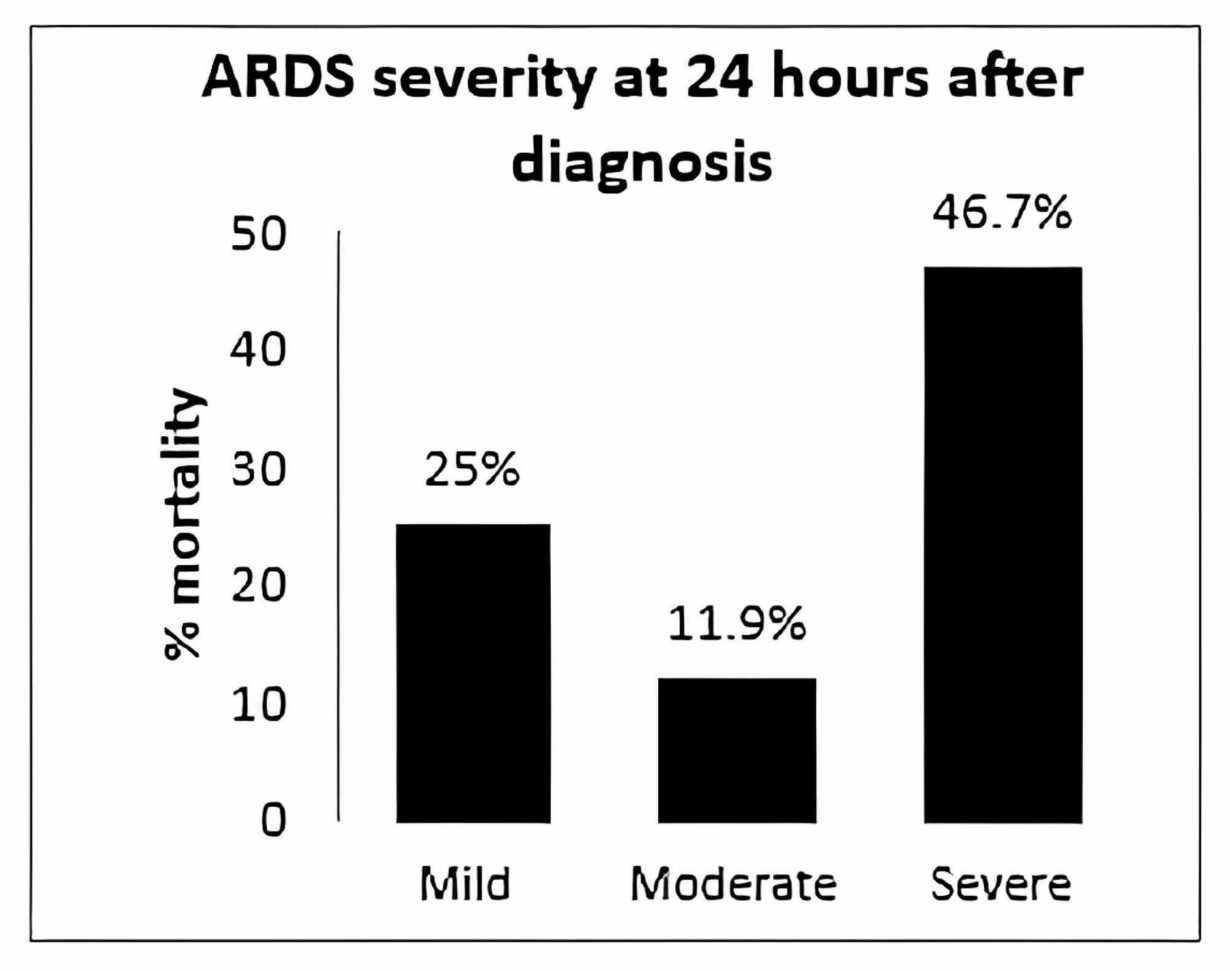
Mortality rate according to ARDS severity at 24 hours after diagnosis (based on the Pa/Fi ratio). ARDS: Acute Respiratory Distress Syndrome, Pa/Fi: Relation between blood pressure and the fraction of inspired oxygen

However, the mortality rates changed 24 hours later - patients with mild, moderate, and severe ARDS had mortality rates of 25%, 11.9%, and 46.7%, respectively (Figure 3).

Another critical consideration in this context was the relation between serum lactate, tidal volume, PEEP, and mortality, at 24 hours of diagnosis of ARDS and the last day of mechanical ventilation (Table 3).

**Table 3:** PEEP values, tidal volume and serum lactate at 24 hours of diagnosis and the last day of mechanical ventilation. PEEP24: Positive End-Expiratory Pressure at 24 hours; TV 24: Tidal volume at 24 hours; LAC 24: lactate at 24 hours; PEEPLD: PEEP the last day of mechanical ventilation; TVLD: Tidal volume the last day of mechanical ventilation; LACLD: lactate the last day of mechanical ventilation. P25: 25th percentile; P50: 50th percentile; p75: 75th percentile.

	Patients who did not die			Patiens who died			P value
	P50	P25	P75	P50	P25	P75	
PEEP24	6	5	8	9	6	10	0.0136
TV24	7.65	6.8	9.075	7.55	6.85	8.15	0.5906
LAC24	0.98	0.7	1.4	1.5	0.97	2.3	0.0026
PEEPLD	5	4	5	7	5	12	0.001
TVLD	8.1	7	9.575	7	6.9	7.6	0.0034
LACLD	1	0.8	1.325	2.35	1.41	4.1	0.0009

At 24 hours of diagnosis and the last day of mechanical ventilation, the PEEP and serum lactate values were higher in patients who died than those who survived. We found a significant difference in PEEP and serum lactate at 24 hours (p = 0.013 vs. 0.002) and a significant difference in PEEP and serum lactate the last day of mechanical ventilation (p = 0.001 vs. 0.009). The present study did not find a significant difference in tidal volume at 24 hours.

**Figure 4:**
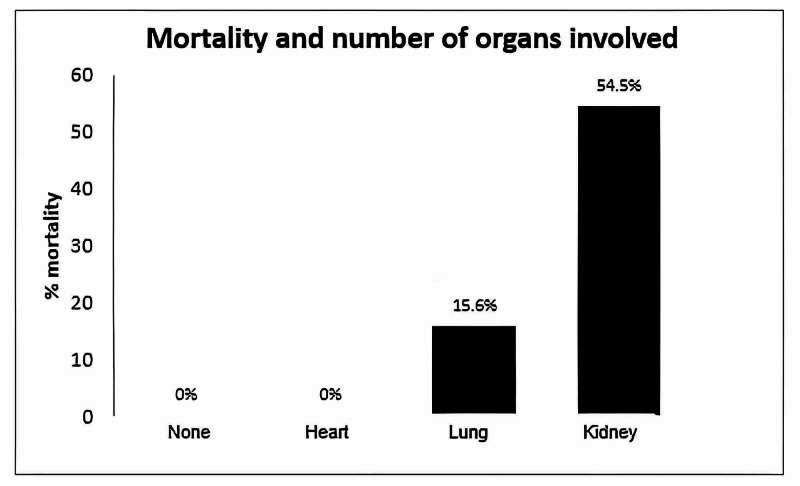
Mortality (%) and number of organs involved.

Concerning the number of organs involved, as the number of involved organs increases, the mortality increases, and kidney dysfunction plays a critical role and is associated with an increased risk for mortality (Figure 4).

## Discussion

The present study is one of the first descriptions of the epidemiology and clinical course of ARDS in pediatric patients at moderate altitude. Reduced oxygen availability on lung physiology is the development of compensatory mechanisms and is an excellent area of academic research interest [7,8,9]. When we used a lung-protective ventilation strategy that has been standardized and studied in pediatric and adult population living at a lower altitude, we did not know the impact in children living at moderate altitudes and diagnosed with ARDS, however, this may affect the clinical course due to the different physiological baseline.

That was the main reason why the research was done in a university hospital of reference. In the present study the leading cause of ARDS was pneumonia (78.1%), followed by bronchiolitis (9%) and sepsis (8%) which is consistent with previous reports in the literature, such as Flori et al. [11], with 328 patients reporting pneumonia as the primary underlying etiology of ARDS in 35% of the cases, followed by aspiration pneumonia and sepsis. In a series of 146 patients in 2012, Lopez et al. [5] confirmed pneumonia as the most common lung condition leading to ARDS.

According to the Pediatric Acute Lung Injury Consensus Conference published in 2015, mechanical ventilation in patients with ARDS should be protective, using tidal volumes between 6 and 8 cc/kg, a maximum plateau pressure of 32 cmH_2_O, and PEEP could be increased up to 10-15 cmH_2_O according to the patient requirements [12].

In our study, these variables were analyzed at admission, 24 hours, day three, day seven, and the last day of mechanical ventilation. We found that the mean of plateau pressure was less than 25.3 cmH_2_O, and tidal volume varied between 7.9 ml/kg and 10.4 ml/kg throughout the study. PEEP was 7.2 cmH_2_O ± 3.2 cmH_2_O; the median was maintained between 6 cmH_2_O and 6.5 cmH_2_O.

With these clinical findings, the correlation between PEEP, tidal volume, and mortality in the first 24 hours, and the last day of mechanical ventilation was estimated. The patients who died had a higher PEEP at 24 hours and the last day of mechanical ventilation (p = 0.013 vs. 0.002), while for the tidal volume, there was no significant difference at 24 hours (p = 0.5) (Table 3). These findings are consistent with the meta-analysis by Petrucci et al. [13], which included six studies performed in adults (1,297 patients) who received mechanical ventilation. The ventilatory strategies were compared, and the first one used a tidal volume of 7 ml/kg or less and a plateau pressure of 30 cmH_2_O or less versus ventilation with tidal volumes between 10 and 15 ml/kg. They reported more significant mortality at 28 days in patients with higher tidal volumes.

The subgroup analysis established that low or intermediate volumes (8-10 ml/kg) could be used if the plateau pressure is not greater than 31 cmH_2_O, which no affect the mortality rate. In the present study, we used intermediate tidal volume and low plateau pressure below 31 cmH_2_O, and it was not correlated with mortality.

These findings have been corroborated in pediatric patients in a meta-analysis and systematic review about tidal volume and mortality in ventilated pediatric patients [14,15], which did not find a relation between tidal volume and mortality. Previous investigations have shown that the overall mortality rate in pediatric ARDS ranges between 35% to 40% [5,11,13].

The severity of ARDS correlates with mortality; however, the Pa/Fi ratio's clinical relevance as a marker of death was not clear. Yehya et al. [14], in a prospective study, determined that the measurement of Pa/Fi at diagnosis was inaccurate because many patients had been reanimated improperly, and this could affect the measurement; at 24 hours the result was much more accurate, and it correlated with mortality. In our study, at diagnosis, the mortality rate was higher in the group with moderate ARDS than in the group with severe ARDS (21.7% vs. 18.8%), however, 24 hours later, the relation was reversed (11.9% vs. 46.7 %).

In 2012 Lopez et al. [5], in a prospective multicenter study, established that mortality correlates with the severity within 24 hours based on the analysis of the PaO_2_/FIO_2_. The patients with severe ARDS patients had a mortality rate of 38.5%, those with moderate ARDS 20%, and those with mild ARDS 11%. Barreira et al. in 2013 conducted a prospective multicenter study in Brazil about the epidemiology and outcomes of pediatric ARDS according to the Berlin definition, and the authors concluded that the group with severe ARDS was the only group with a significant difference in mortality (11% vs. 3%, p = 0.002) and days free of mechanical ventilation (five vs. 20 days, p = 0.001) versus the group with mild and moderate ARDS [15].

Our study findings are partially correlated, the mortality rate in patients with severe ARDS was 46.7% at 24 hours; however, the patients' group with mild ARDS had higher mortality than the moderated ARDS at 24 hours. This could be explained by the fact that Pa/Fi can be modified by the ventilation parameters and the vasoactive drug support; in this way, the patients classified as moderate at diagnosis, 24 hours later were classified as mild, then on day three, day seven, and last day of mechanical ventilation the mild ARDS turns into a severe ARDS and dies. These findings make us believe that Pa/Fi is not a good predictor of mortality, and there are more factors involved in the outcome.

Several independent research groups [13,14,15,16] have shown that the number of involved organs increases linearly as higher ARDS severity increases. Our study is correlated with previously published findings. The heart and lung compromise did not increase the mortality rate in our study, but if there is also kidney compromise, the mortality increases up to 15% and increases up to 54.5% if there are more than four organs involved, including heart, lung, kidney and hematologic (Figure 4).

The main limitation of this study is that it is not possible to directly extrapolate our results about mortality to the entire pediatric population living at a moderate altitude above mean sea level because this study was conducted in one single medical institution.

## Conclusions

This study shows that pediatric ARDS clinical behavior in patients at moderate altitudes is similar to those pediatric patients with ARDS at low altitudes.

All patients had the same lung-protective ventilation, and it was not necessary to increase the ventilator parameters. The goals of oxygenation and alveolar ventilation were achieved. Tidal volume is an independent variable for mortality, while serum lactate and PEEP are correlated with mortality. We also identified the Pa/Fi as a poor predictor of mortality for patients with mild or moderate ARDS because Pa/Fi can be modified by the ventilator parameters and vasoactive drug support, and it was not correlated with mortality at 24 hours. Further multicenter studies are needed to assess the current outcome.
